# Distribution analysis of epertinib in brain metastasis of HER2-positive breast cancer by imaging mass spectrometry and prospect for antitumor activity

**DOI:** 10.1038/s41598-017-18702-2

**Published:** 2018-01-10

**Authors:** Yukari Tanaka, Michinari Hirata, Satomi Shinonome, Mikinori Torii, Ken-ichi Nezasa, Hidekazu Tanaka

**Affiliations:** 1 Drug Metabolism and Pharmacokinetics, Research Laboratory for Development, Shionogi & Co., Ltd., Toyonaka, Osaka, Japan; 2 Oncology and Immunology, Drug Discovery and Disease Research Laboratories, Shionogi & Co., Ltd., Toyonaka, Osaka, Japan; 3 Drug Safety Evaluation, Research Laboratory for Development, Shionogi & Co., Ltd., Toyonaka, Osaka, Japan

## Abstract

Epertinib (S-222611) is a potent, reversible, and selective tyrosine kinase inhibitor of epidermal growth factor receptor (EGFR), human EGFR2 (HER2), and human EGFR4. We developed experimental brain metastasis models by intraventricular injection (intraventricular injection mouse model; IVM) of HER2-positive breast cancer (MDA-MB-361-luc-BR2/BR3) or T790M-EGFR-positive lung cancer (NCI-H1975-luc) cells. After a single oral administration, epertinib and lapatinib concentrations in brain metastatic regions were analyzed by quantitative imaging mass spectrometry. In the NCI-H1975 lung cancer IVM, the concentration of epertinib in brain metastasis was comparable to that of lapatinib. However, in the MDA-MB-361 breast cancer IVM, the concentration of epertinib in brain metastasis was >10 times higher than that of lapatinib. Furthermore, the epertinib tumor-to-normal brain ratio was ~4 times higher than that of lapatinib. Blood-tumor barrier (BTB) permeability was assessed in each brain metastatic region. In the lung cancer model, fluorescently labeled dextran was more highly detected in brain metastatic regions than in brain parenchyma. However, in breast cancer models, dextran fluorescence intensity in brain metastatic regions and brain parenchyma were comparable, suggesting that the BTB remained largely intact. Epertinib would be promised as a therapeutic agent for HER2-positive breast cancer with brain metastasis.

## Introduction

Breast cancer is a heterogeneous disease with treatment options varying with biological markers. Individual patient prognosis depends on the status of biological markers in the primary tumor including the estrogen receptor (ER), progesterone receptor (PR), human epidermal growth factor receptor 2 (HER2), and Ki67^1,2^. HER2 is amplified in 25–30% of human breast cancers and is associated with enhanced tumor aggressiveness and reduced patient survival^3,4^. Recently, the rate of brain metastasis of breast cancer has increased, occurring in ≥15% of patients. In some patient sub-populations, with tumors defined as HER2-positive or “triple-negative” (ER and PR negative, HER2 negative), the rate of brain metastasis exceeds 35%^5–7^. Patients with HER2-positive and triple-negative breast cancer are at increased risk of developing brain metastasis^6,8^. Longer survival of patients with metastatic breast cancer and improved imaging techniques are associated with the increased incidence of reported brain metastasis. Patients who develop brain metastases tend to have poor prognosis with short overall survival. Additionally, brain metastasis is a major cause of morbidity, and is associated with progressive neurological deficits resulting in reduced quality of life^5–8^. Current therapies for patients with breast cancer brain metastasis include surgical resection, whole-brain radiation therapy, stereotactic radiosurgery, chemotherapy, and targeted therapy^9^. However, the therapeutic benefits of these therapies are limited and unmet medical needs remain. The development of brain metastasis is complex and, requires invasion of primary breast cancer cells into surrounding tissues and vessels, traffic through the circulatory system, colonization, and growth in the brain parenchyma. Furthermore, many available therapies are unable to cross the blood-tumor barrier (BTB)^10^.

Lapatinib, a small molecule EGFR and HER2 dual kinase inhibitor, was the first agent approved by the FDA for the treatment of advanced or metastatic breast cancer^11^. Lapatinib has been extensively tested in the treatment of breast cancer patients with brain metastases and clinical information, including clinical pharmacokinetic (PK)/pharmacodynamics (PD) studies^12^. Additionally, it is possible to evaluate lapatinib uptake in experimental brain metastases models. Using clinically relevant doses of lapatinib, it is possible to compare the distribution in clinical and non-clinical studies. The FDA has approved the clinical use of lapatinib in combination with capecitabine (Xeloda^®^). However, to accurately evaluate the single drug distribution, we selected a single administration of lapatinib as the reference experimental setting.

Epertinib (S-222611) is an oral, reversible EGFR, HER2, and HER4 tyrosine kinase inhibitor (TKI) with antitumor activity in animal models expressing these proteins^13^. In phase I trials, epertinib has been well tolerated with efficacy against HER2-positive tumors, including breast cancer metastasized to brain^14–17^. In phase I/II trials, the progression free survival of patients treated with epertinib is longer than that reported in other studies of TKIs for which a similar target population was enrolled. Furthermore, treatment with epertinib, in combination with trastuzumab, showed a tumor response in seven of 21 patients previously treated with lapatinib in combination with capecitabine or ado-trastuzumab emtansine^18^. In all the participants in a clinical trial, two of 45 patients with brain metastases as the target lesion showed tumor reduction (partial response). To understand the mode of action of epertinib in patients with intact BTBs, we developed experimental brain metastasis models in mice by serial *in vivo* passage (intraventricular injection mouse model; IVM). Mechanistic understanding of pharmacological and toxicological events *in vivo* can fundamentally improve the chances of a candidate compound succeeding in a clinical trial. Many analytical tools aim to examine the *in vivo* distribution of a drug, and its metabolites, in target tissues. Liquid chromatography-tandem mass spectrometry (LC-MS/MS) with electrospray ionization is widely used in drug distribution studies^19,20^. However, LC/MS/MS cannot provide spatial information on drug distribution within an organ because the analyte is extracted for quantification from homogenized tissue samples.

Matrix-assisted laser desorption/ionization imaging mass spectrometry (MALDI-IMS) was developed to directly visualize the distribution of small (drugs, lipids, and endogenous metabolites) and large molecules (peptides and proteins) in tissue sections without radiolabeling^21–23^. The MALDI-IMS method has been developed for quantification of small molecule drugs in the past few years^24–26^. Quantitative MALDI-IMS has been applied to gain an understanding of the molecule-based distribution of pharmacological agents in heterogeneous tissues with complex structures^27,28^. To understand the efficacy and safety of drug candidates, it is important to know both the specific distribution and concentration of a compound within the target regions.

Here, we aimed to enhance the clinical predictability of epertinib by evaluating its distribution and efficacy in the treatment of brain metastasis of HER2-positive breast cancer. We used HER2-positive breast cancer, or T790M-EGFR expressing lung cancer IVMs. Additionally, we employed quantitative MALDI-IMS to analyze the distribution and concentration of epertinib in brain metastasis and compared the results with those of competitive agent. Our results show that MALDI-IMS can be utilized in practical applications in drug discovery research.

## Results

### Pharmacokinetics of epertinib and lapatinib in mice

**Figure 1:**
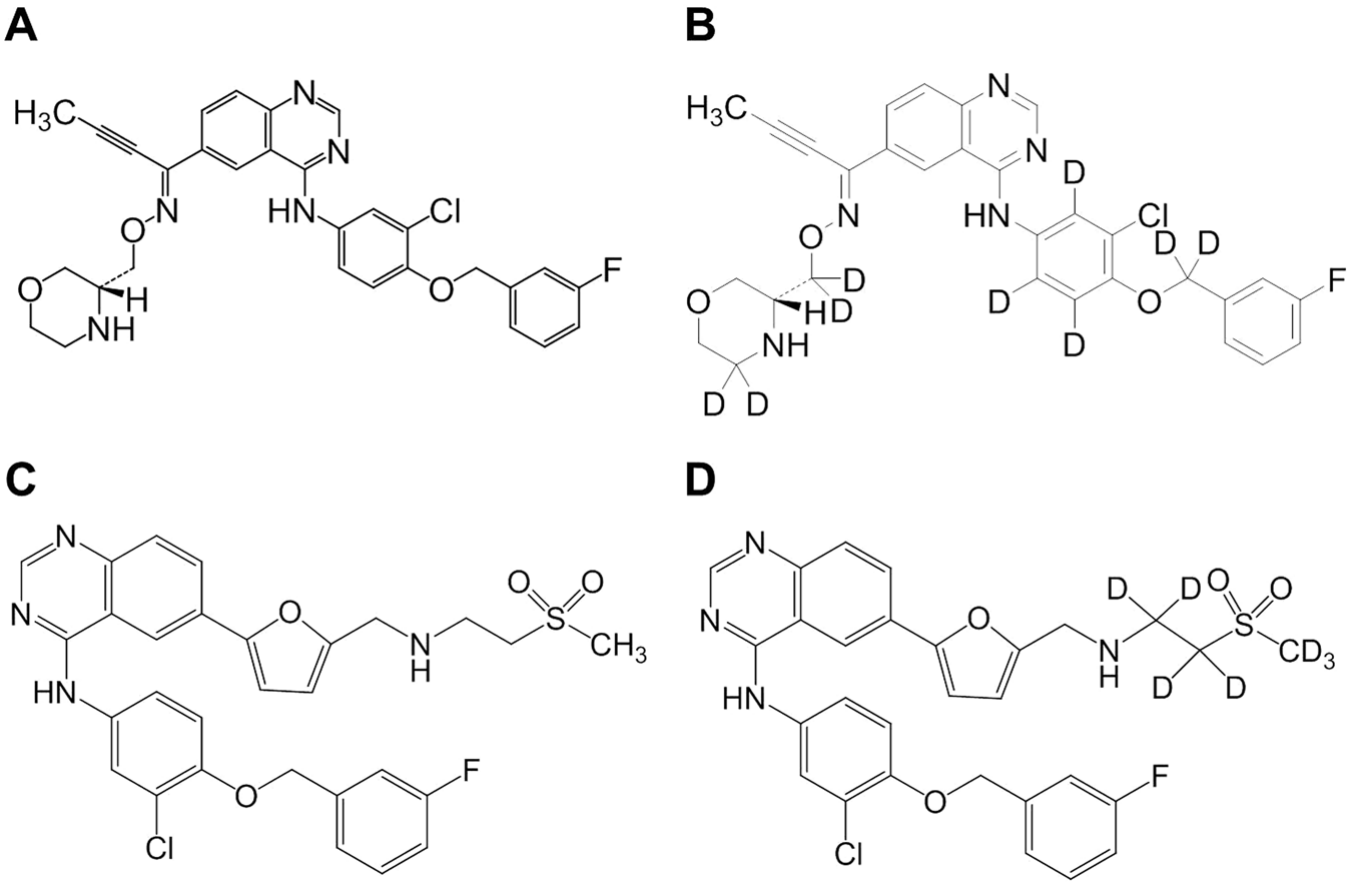
Chemical structures of epertinib (**A**), epertinib-*d*_9_ (**B**), lapatinib (**C**), and lapatinib-*d*_7_ (**D**).

In order to support the effects of epertinib and lapatinib on brain metastasis from a perspective of pharmacokinetics, plasma and brain concentrations of epertinib and lapatinib are shown in Table 1. The pharmacokinetic parameters observed after single oral administrations of epertinib hydrochloride or lapatinib ditosylate monohydrate (20 mg/kg as epertinib or lapatinib) in mice implanted intracranially with MDA-MB-361 are shown in Table 1. The chemical structures of epertinib, epertinib-*d*_9_, lapatinib, and lapatinib-*d*_7_ are shown in Fig. 1. T_max_ values of both epertinib and lapatinib in brain were achieved 4 h after oral administration. Four hours after oral administration, the brain concentration of epertinib was approximately twice that of lapatinib. The plasma exposure (AUC_0–24 h_) after a single oral administration of 50 mg/kg epertinib (9.98 µg·h/mL) or lapatinib (34.1 µg·h/mL) in nude mice was equivalent to that of epertinib (9.17 µg·h/mL) and lapatinib (27.3 µg·h/mL) in human at the effective doses of 800 mg/day or 1250 mg/day, respectively^17^.

**Table 1:** Comparison of plasma and brain concentrations, and pharmacokinetic parameters between epertinib and lapatinib after single oral administration at 20 mg/kg in mice implanted intracranially with MDA-MB-361 cells.

Time (h)	Epertinib		Lapatinib	
	Plasma	Brain	Plasma	Brain
	(ng/mL)	(ng/g)	(ng/mL)	(ng/g)
0.5	99.8	10.2	984.5	11.8
1	112.9	12.8	762.8	15.7
2	145.0	23.0	413.4	10.0
4	114.8	36.3	461.1	17.0
8	54.7	23.8	99.6	5.8
24	2.1	1.7	5.4	0.4
Cmax (ng/mL or ng/g)	145	36	985	17
Tmax (h)	2.0	4.0	0.5	4.0
AUC_0–24 h_ (ng·h/mL or ng·h/g)	1261	409	4107	144

Concentration data represents the mean of five mice except for 24 h data of lapatinib. (n  =  4 for the 24 h data of lapatinib).

### Distribution of epertinib and lapatinib in brain metastases of the breast cancer IVM

**Figure 2:**
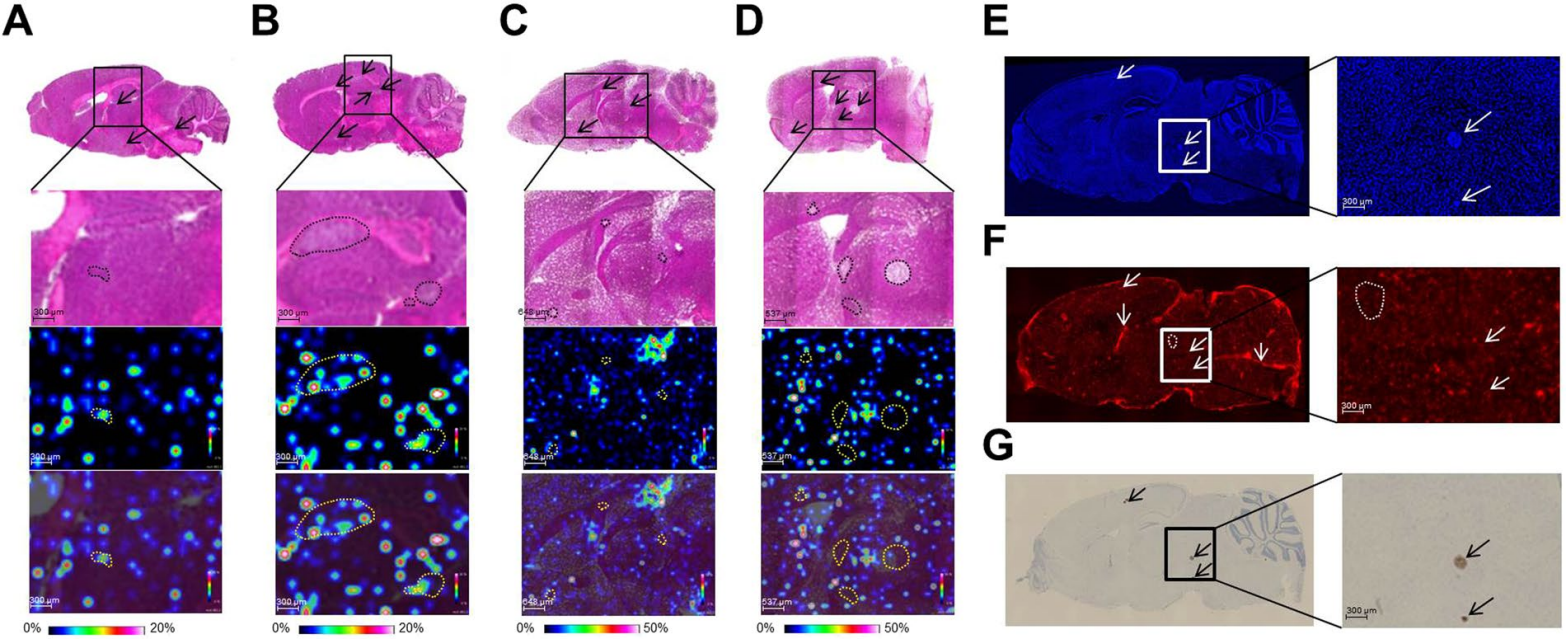
Distribution of epertinib and lapatinib in brain metastases of the breast cancer IVM. Representative images of mouse brain sections showing BR3 IVM with breast cancer with at 4 h (**A**) and 8 h (**B**) after oral administration of epertinib hydrochloride at a dose of 50 mg/kg (as epertinib), and BR3 IVM with breast cancer at 4 h (**C**) and 8 h (**D**) after administration of lapatinib ditosylate monohydrate (as lapatinib, 50 mg/kg). The magnified views are shown in upper row: H&E images, middle row: ion images of epertinib or lapatinib, and lower row: ion images of epertinib or lapatinib co-registered with H&E. Typical images of mouse brain sections showing diamidino phenylindole fluorescence (**E**), Texas-Red^®^ dextran (3 kDa) fluorescence (**F**), and HER2 IHC staining (**G**) from BR3 IVM of breast cancer with brain metastases. Arrows indicate the position of tumors and the position of choroid plexus. Dashed lines of black and yellow: the region of tumors (**A–D**), white: the region of brain parenchyma (**F**).

Based on the pharmacokinetic studies of epertinib and lapatinib in mouse and human, we performed a single oral dosing of 50 mg/kg epertinib or lapatinib in the IVMs (epertinib: BR2 and BR3 euthanized 4 h post dose administration: n  =  3, BR3 euthanized 8 h post dose administration: n  =  3, lapatinib: BR2 and BR3 euthanized 4 h post-dose administration: n  =  3, BR3 euthanized 8 h post-dose administration: n  =  2). Prior to IMS analysis, H&E staining was performed on serial sections to identify the position of tumors in the brain sections, and multiple small tumors (criteria: number of tumors  >  2) were observed in the brains of the models. Epertinib and lapatinib distribution in tumors was visualized by IMS using the brain sections of the IVMs, and is clearly illustrated in magnified displays overlaying the ion and H&E staining images (Fig. 2). At 4 and 8 h after drug administration, epertinib signal was clearly detected by IMS in BR3 tumors (Fig. 2A and B) and in BR2 IVM tumors 4 h after administration (Supplementary Fig. S2A). However, lapatinib signal was quite low in tumors of both IVMs (Fig. 2C and D, and Supplementary Fig. S2B). BTB integrity in the IVM was assessed by measuring the fluorescence intensity of Texas-Red^®^ dextran in the metastatic regions or brain parenchyma. Metastatic regions were defined as cancer cell clusters with DAPI staining in the same brain section (Fig. 2E and Supplementary Fig. S2C). Fluorescence intensity in the tumor and brain parenchymal regions was comparable, but lower than that observed in the choroid plexus (Fig. 2F and Supplementary Fig. S2D). The BTB remained largely intact in brain metastases of the IVM with breast cancer.

HER2 IHC was performed using serial sections to analyze the levels of HER2 expression in individual metastatic regions. HER2 expression was observed in all brain metastases of the IVM with breast cancer, and HER2 expression levels varied little among brain metastases (Fig. 2G and Supplementary Fig. S2E).

### Quantitative analysis of epertinib and lapatinib

**Figure 3:**
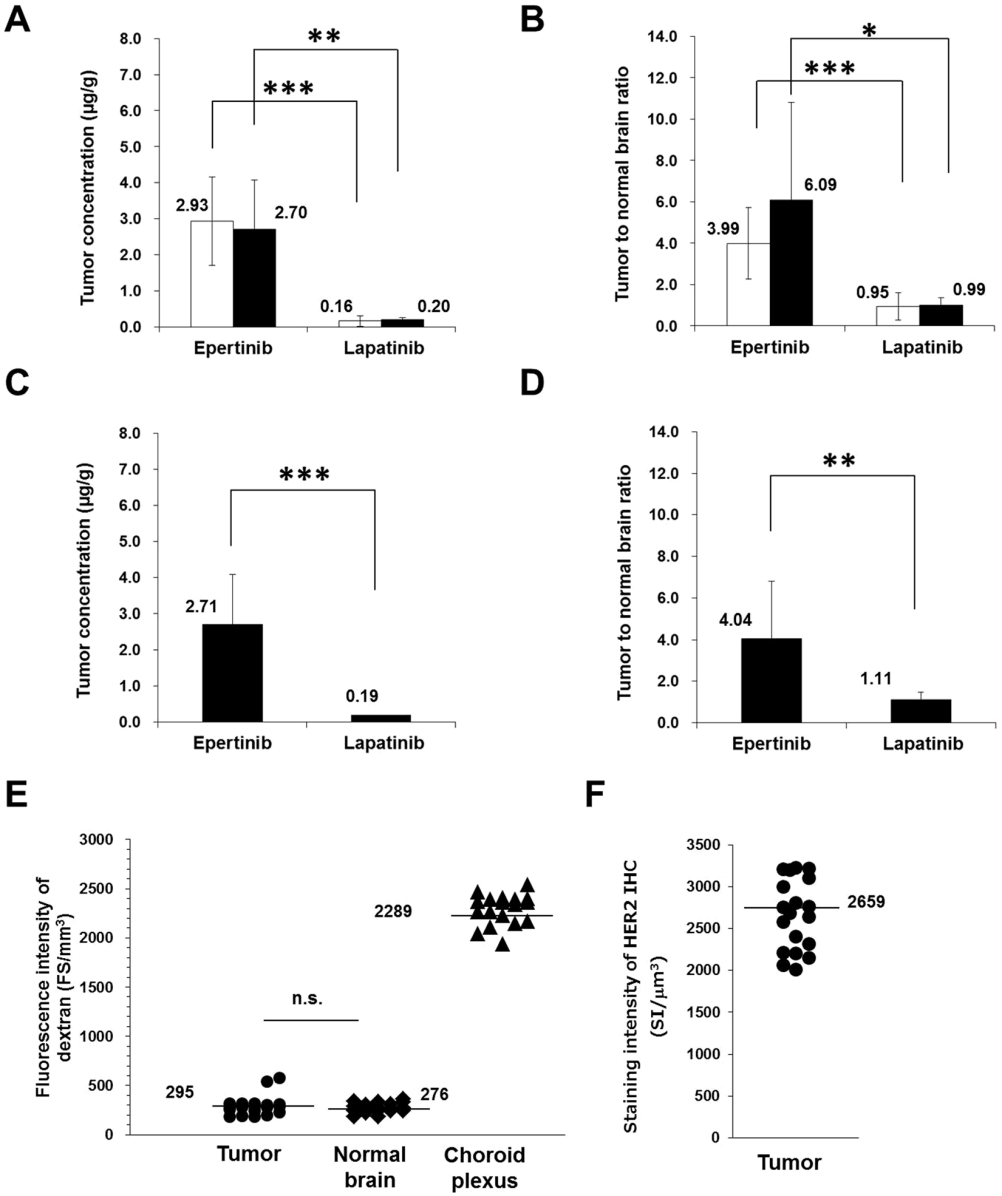
Quantitative analysis of epertinib and lapatinib in IVMs of breast cancer by IMS. The tumor concentration and tumor-to-normal brain ratio at 4 h after oral administration of epertinib or lapatinib in IVMs of breast cancer with MDA-MB-361-luc-BR2 (BR2) (open bars) and MDA-MB-361-luc-BR3 (BR3) (filled bar) are shown in (**A**) and (**B**) (epertinib: BR2 (n  =  19), BR3 (n  =  8), lapatinib: BR2 (n  =  25), BR3 (n  =  11)). At 8 h after oral administration of epertinib or lapatinib in BR3 IVM, the tumor concentration and tumor-to-normal brain ratio are shown in (**C**) and (**D**) (epertinib: n  =  15, lapatinib: n  =  12). The results of statistical analysis (*P  <  0.05, **P  <  0.01, ***P  <  0.001, epertinib versus lapatinib, Welch’s t-test) are presented in (**A–D**). Mice (n  =  6) were sacrificed at 4 h after administration epertinib or lapatinib. Each point represents fluorescence intensity for each lesion of brain metastases (n  =  20) of 6 mice and those for areas of normal brain (n  =  18) and choroid plexus (n  =  18), (3 points randomly selected for each mouse), (**E**). Each point shows staining intensity of HER2 immunohistochemistry (n  =  19), (**F**) (n.s.: not significant).

To determine the concentrations of epertinib and lapatinib in tumors of brain sections by IMS, the standard solutions used for calibration curves were directly spotted near the brain sections, on the same glass slides. Calculations were performed after normalization to the signal of epertinib-*d*_9_ or lapatinib-*d*_7_ signal as the internal standards to minimize ion suppression among the regions of the brain sections and between the tissue surface and glass slides^31,32^. In order to confirm the robustness of the IMS measurement, each brain section was determined between the calibration curves, resulting that the slopes of the calibration curves were almost the same and the correlation coefficients were over 0.99 (Supplementary Fig. S4). Four hours after administration, the tumor concentration of epertinib was over 10 times higher than that of lapatinib, and the tumor-to-normal brain ratio of epertinib was approximately four times higher than that of lapatinib in both IVMs (Fig. 3A and B). Eight hours after administration, the tumor concentration of epertinib in the IVM was maintained, and the tumor-to-brain ratio of epertinib was significantly higher than that of lapatinib (Fig. 3C and D). The plasma concentration of epertinib 4 h (1.44  ±  0.05 µg/mL and 1.30  ±  0.12 µg/mL for BR2 and BR3, respectively) and 8 h (0.92  ±  0.30 µg/mL for BR3) after drug administration in the IVM was measured by LC-MS/MS. Similarly, the plasma concentration of lapatinib 4 h (6.69  ±  1.31 µg/mL and 2.14  ±  0.22 µg/mL for BR2 and BR3, respectively) and 8 h (0.63 µg/mL for BR3) after drug administration was determined by LC-MS/MS. The plasma concentrations of both compounds in the IVM were lower at 8 h after administration than at 4 h after administration.

The average dextran fluorescence intensity observed in brain metastases (n  =  20) and in normal brain regions (n  =  18) was comparable. In brain sections (n  =  18), the average dextran fluorescence intensity in metastatic regions was 7.8-fold less than the average intensity observed in the choroid plexus (Fig. 3E). HER2 IHC revealed only a 1.7-fold difference between brain metastatic regions (n  =  19), indicative of low heterogeneity of HER2 expression in the brain metastases of the IVM with breast cancer (Fig. 3F).

### Quantitative distribution analysis of epertinib and lapatinib in brain metastases of the lung cancer IVM

**Figure 4:**
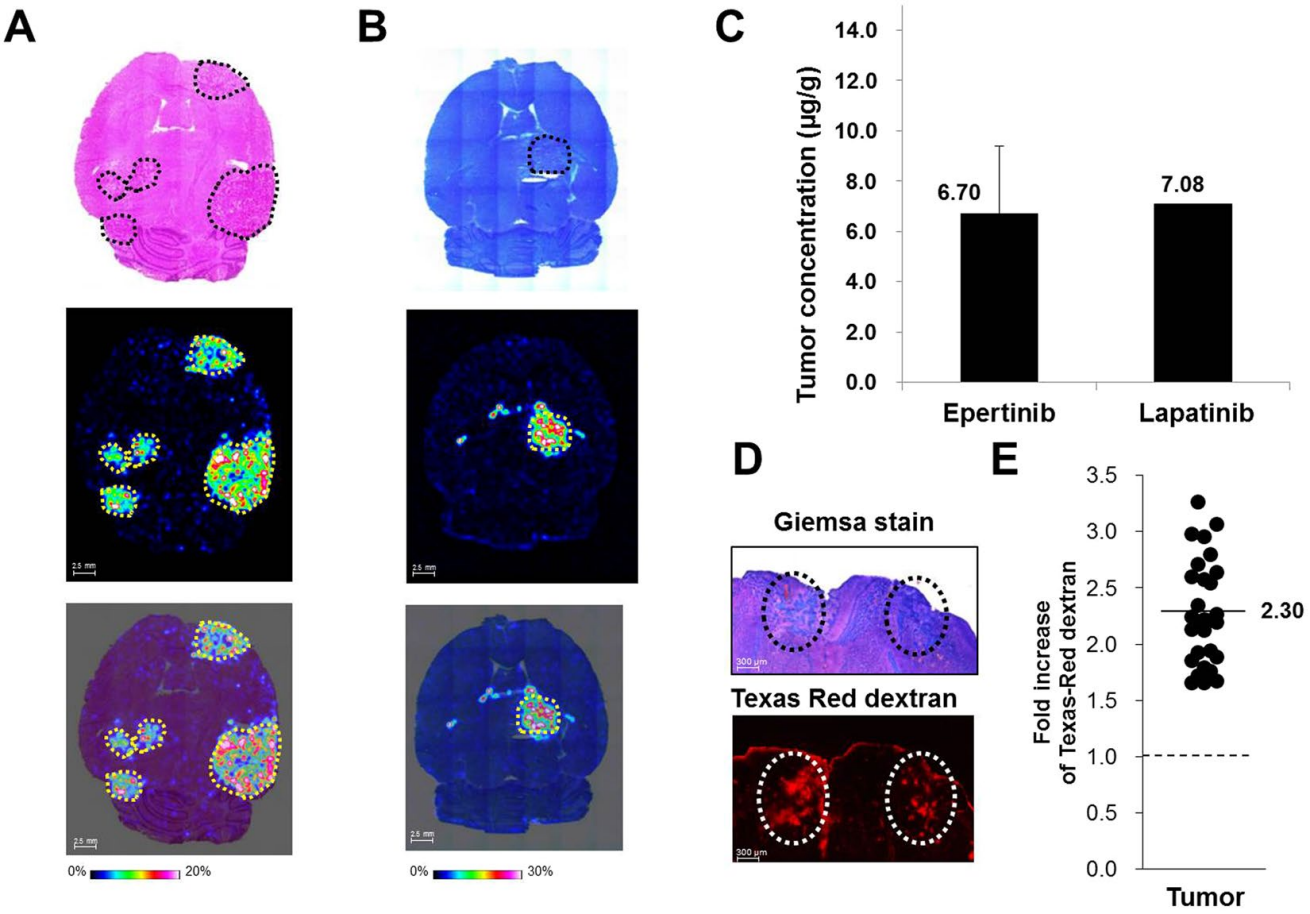
Quantitative distribution analysis of epertinib and lapatinib in brain metastases of the lung cancer IVM. Representative images of horizontal brain sections of mice showing IVM with lung cancer (NCI-H1975-luc) at 4 h after oral administration of epertinib hydrochloride or lapatinib ditosylate monohydrate at a dose of 100 mg/kg (as epertinib (**A**) or lapatinib (**B**)). The ion images of epertinib (**A**) or lapatinib (**B**) are shown in middle row. In lower row, the ion images of epertinib (**A**) or lapatinib (**B**) are overlaid on the stained images ((A): H&E staining, (B): giemsa staining). The tumor regions are enclosed by dashed yellow lines (**A** and **B**). The tumo29r concentration at 4 h after oral administration of epertinib or lapatinib in IVM with lung cancer are shown in (**C**) (epertinib: (n  =  13), lapatinib (n  =  2)). Typical images of mouse brain sections showing giemsa staining (upper) and Texas-Red^®^ dextran (3 kDa) fluorescence (lower) from IVM with NCI-H1975-luc brain metastases. Circles indicate the position of tumors (**D**). Each point represents fold increase of Texas-Red^®^ dextran (3 kDa) fluorescence intensity for each region of brain metastases (n  =  29) of 18 mice and those for areas of normal brain (n  =  3) (**E**).

The concentrations of epertinib and lapatinib in brain metastasis of the IVM with lung cancer were analyzed by IMS. The staining was performed on serial sections to identify the positions of tumors in the brain sections, and indicated that the tumor size in brain of the IVM with lung cancer was larger than that observed in the IVM with breast cancer. This difference was attributed to the faster growth of NCI-H1975-luc cells (*in vivo* doubling time was 26 h and 176 h for NCI-H1975-luc and BR3, respectively). This is clearly illustrated in ion images showing the overlay of the epertinib or lapatinib ion and staining images. Four hours after administration of 100 mg/kg epertinib or lapatinib, the signal intensity of both compounds in tumors of the IVM with lung cancer were detected by IMS (Fig. 4A and B). Quantitative IMS analysis revealed that the tumor concentration of epertinib in the brain of the IVM with lung cancer was equivalent to that of lapatinib in the same tissues (Fig. 4C). In the IVM with lung cancer, the plasma concentrations of epertinib and lapatinib were 2.76  ±  0.66 µg/mL and 9.36  ±  1.78 µg/mL, respectively, 4 h after administration. The study on dextran incorporation showed that the average fluorescence intensity in brain metastasis (n  =  29) was 2.3-fold higher than the average intensity in normal brain regions (n  =  3), indicating that the BTB was disrupted in the lung cancer model. Tissue drug uptake in the IVM with lung cancer occurred irrespective of the physicochemical properties of the drug due to the disruption of the BTB.

### Co-localization analysis of epertinib and the heme B blood marker in brain of IVMs with breast cancer

**Figure 5:**
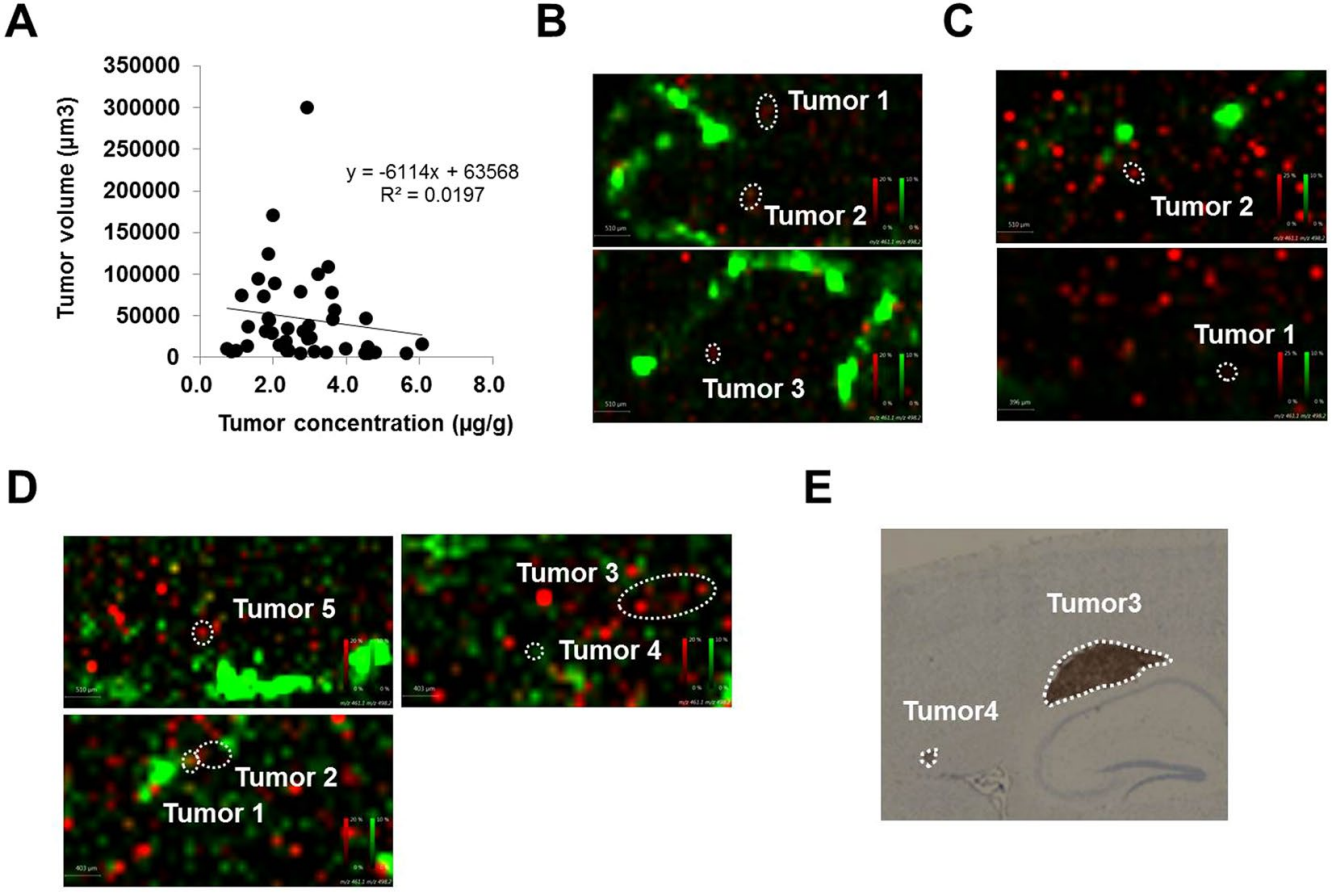
Co-localization analysis of epertinib and heme B in brain of IVMs with breast cancer. Correlation analysis between the tumor epertinib concentration and the tumor volume (µm^3^), r^2^  =  0.02, number of metastases: n  =  42, (**A**). Co-localization of epertinib (red) and heme B (green) in the brain sections of IVM with breast cancer (**B** and **C**), tumor concentration of epertinib: (**B**) tumor 3  >  tumor 1 ≈ tumor 2, (**C**) tumor 2  >  tumor 1. In the BR3 IVM sectioned brain, at 8 h after epertinib administration, only tumors 3 and 4 showed no correlation between tumor epertinib concentration (tumor 5  >  tumor 1  >  tumor 2, tumor 3  >  tumor 4) and the ion intensity of heme B (**D**). The HER2 IHC image observed in tumor 3 and 4 in BR3 IVM with breast cancer is shown in (**E**).

The tumor concentration of epertinib varied several-fold within brain sections and between individuals. Analysis of the correlation between the tumor epertinib concentration and the tumor volume (µm^3^) (tumor volume  =  tumor area  ×  section thickness) revealed a weak correlation (r^2^  =  0.02, number of metastases: n  =  42) (Fig. 5A). Next, we assessed the co-localization of epertinib and the heme B, blood marker, in the brain of the IVMs with breast cancer using IMS^33,34^. In most brain sections of the IVM with breast cancer, the ion intensity and pixel area of heme B around the tumors increased with increasing tumor concentrations of epertinib (Fig. 5B: tumor 3  >  tumor 1 ≈ tumor 2 and 5 C: tumor 2  >  tumor 1). In the BR3 IVM sectioned brain, harvested 8 h after epertinib administration, only tumors 3 and 4 showed no correlation between tumor epertinib concentration and ion intensity and pixel area of heme B (Fig. 5D: tumor 5  >  tumor 1  >  tumor 2, tumor 3  >  tumor 4). The HER2 expression level observed in tumor 3 was notably higher than that observed in tumor 4 (Fig. 5E).

### Antitumor activities of epertinib in the experimental brain metastasis of HER2-positive breast cancer

**Figure 6:**
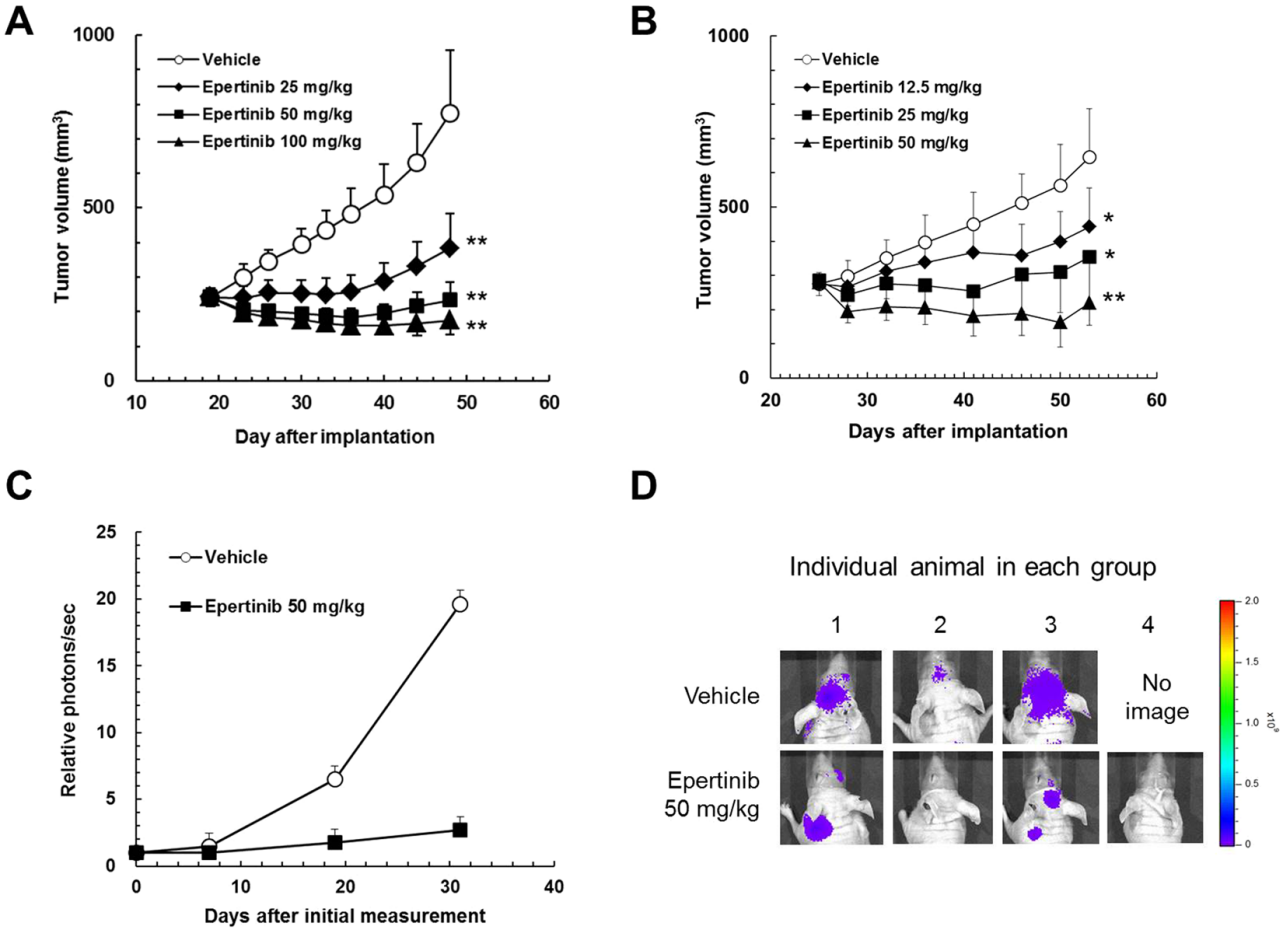
*In vivo* antitumor activity of epertinib in the breast cancer IVM. Epertinib inhibits the growth of breast cancer in animal models. In each animal model, vehicle or indicated doses of epertinib were orally administered daily. Tumor volume was measured twice or thrice weekly and the mean tumor volume with SD is represented by each data point. Human breast cancer cells, MDA-MB-361 (**A**) or MDA-MB-361-luc-BR2 (**B**) was implanted orthotopically into the mammary fat pad of mice. Tumor-bearing mice were treated for 28 days (**A** and **B**). Tumor growth in the breast cancer IVM (MDA-MB-361-luc-BR3) was monitored by the photons emitted from the tumor as an indicator, which was measured with an IVIS Imaging System 200 (Caliper Life Sciences). Tumor-bearing mice were orally administered vehicle or the 50 mg/kg doses of epertinib for 30 days. The photons were measured once or twice weekly and the mean photons/sec with SD are presented at each data point (n  =  3 for vehicle, n  =  4 for 50 mg/kg of epertinib) (**C**). Bioluminescent images at the last measurement *P  <  0.05, **P  <  0.01 (epertinib versus vehicle, Dunnet’s test) (**D**).

We evaluated the inhibitory activity of epertinib on the *in vitro* growth of the parental cell line, MDA-MB-361. The epertinib IC_50_ value for the MDA-MB-361 cell line was 26.5 nmol/L, remarkably lower than the concentration (5.2 µmol/L) of epertinib measured in brain metastases of the IVM with breast cancer. To compare the growth inhibitory activity of epertinib on the parental and metastatic (BR2) cell lines, we evaluated the antitumor activity of epertinib in both cell lines. Epertinib showed antitumor activity in the mammary fat pad implantation model using both cell lines and the ED_50_ values were comparable (24.1 mg/kg and 26.5 mg/kg for MDA-MB-361 and BR2, respectively, Fig. 6A and B). To confirm the antitumor activity of epertinib in the IVM, we examined the effect of epertinib on the IVM transplanted BR2. Once daily oral administration of 50 mg/kg epertinib significantly reduced the brain tumor volume, indicating that epertinib could have potent antitumor activity in brain metastasis even in the presence of an intact BTB (Fig. 6C and D).

## Discussion

We used quantitative IMS to analyze and compare the distribution of epertinib and lapatinib in brain metastasis. IMS can simultaneously detect diverse molecules and obtain spatial distribution information capable of distinguishing between a drug and its metabolites in tissue sections^21,35^. Historically, drug metabolism and pharmacokinetic studies have used radiolabels to perform autoradiography and obtain information about tissue distribution. Previously, plasma concentrations of radioactivity and epertinib were determined after a single oral administration of [^14^C]-epertinib hydrochloride at a dose of 5 mg/kg (as epertinib) in rats (Supplementary Fig. S3). The plasma concentration of radioactivity decreased from a maximum (C_max_) of 93.4  ±  16.0 ng eq./mL at 4.00  ±  1.63 h after dosing. The area under the blood concentration-time curve (AUC)_0–48 h_ and AUC_inf_ values were 1720  ±  290 and 1870  ±  330 ng eq.·h/mL, respectively. The mean plasma concentrations of epertinib declined from a C_max_ of 14.1  ±  3.6 ng/mL at 5.00  ±  1.15 h after dosing. The AUC_0–12 h_ and AUC_inf_ values were 126  ±  18 and 392  ±  316 ng·h/mL, respectively. The plasma epertinib/radioactivity concentration ratio ranged from 10.9% to 16.2% through the measurable points, and the AUC_inf_ ratio was ~20.8  ±  15.0%. These results suggested that [^14^C]-epertinib was rapidly metabolized after oral administration and the majority of radioactivity in plasma was attributed to epertinib metabolites. The plasma concentration-time profiles of total unchanged lapatinib and radioactivity were measured in healthy volunteers after a single oral administration of 250 mg of [^14^C]-lapatinib^36^. The mean plasma concentration of lapatinib and total radioactivity at C_max_ were 330 ng/mL and 720 ng eq./mL, respectively. In this study, autoradiography could not be used to analyze the distribution of epertinib and lapatinib in brain metastasis of the IVM because of the confounding effects of epertinib and lapatinib metabolites produced *in vivo*.

IMS allows the analysis of the distribution and concentration of drugs in small and complicated structures, such as brain metastases of the IVM. We previously developed a novel quantitative IMS method for small molecule drugs, making it possible to normalize ion suppression of a drug in some tissues using internal standards such as stable isotope labeling and structural analogs^32^. The quantitative capability of MALDI-IMS has recently been recognized and used in several studies applying quantitative IMS to drug discovery research^37,38^. The concentrations of epertinib in brain metastasis of both IVMs were comparable. Reproducibility of epertinib pharmacodynamics in the IVM using different cancer cell lines, combined with the results of the IMS study, indicates that epertinib can migrate to brain tumors resulting from breast cancer metastasis. In the IVM with lung cancer, the migration of epertinib to brain metastasis was rarely different from that of lapatinib. However, the migration of epertinib to brain metastases was significantly higher than that of lapatnib in the IVM with breast cancer. Dextran fluorescence was more intense in brain metastases than in parenchyma in the IVM with lung cancer. In the breast cancer IVM, the fluorescence intensity of dextran in brain metastasis and parenchyma were comparable. These results indicate that the BTB remained largely intact in the breast cancer model but was disrupted in the lung cancer model. Maintenance of BTB integrity in the breast cancer model was confirmed by the tumor-to-normal brain ratio of lapatinib. These findings indicate that the migration of epertinib into brain tumors caused by breast cancer metastasis is superior to that of lapatinib.

We developed the experimental brain metastasis of HER2-positive breast cancer MDA-MB-361 by serial *in vivo* passages. MDA-MB-361 has been originally established from brain metastasis site of breast cancer patient, and known as highly expressed endogenous HER2^39^. BTB heterogeneity has been shown in the exogenous HER2 overexpressing MDA-MB-231-BR-HER2 experimental brain metastasis model, and lapatinib distribution varied among brain metastases, correlating with altered BTB permeability^40^. Brain tumor sizes in our IVM were smaller and BTB permeability was largely unchanged, indicating that our system behaves similarly to the early phase of brain metastasis. Most anti-cancer drugs have limitations in drug delivery, caused by ATP-binding cassette (ABC) efflux transporters, P-glycoprotein (P-gp) and breast cancer resistance protein (BCRP), expressed at the BTB^41^. We examined the permeability and efflux ratio of epertinib across Caco-2 cell monolayers and found that epertinib is not a substrate of P-gp and BCRP (Supplementary Table S1). However, lapatinib, neratinib, and afatinib TKIs are P-gp and BCRP substrates^42–44^, as is tucatinib (ONT-380), a HER2-selective inhibitor of small molecules^45^.

The protein binding of epertinib in mouse, rat, dog, and human plasma have been studied. The binding ratios of epertinib at concentrations of 0.1, 1, and 10 µg/mL were ≥99.8%, 99.4%, 99.6%, and 99.5% in mouse, rat, dog, and human plasma, respectively, indicating that no difference in the binding of epertinib to plasma in the species analyzed. Lapatinib has a high binding affinity (>99.9%) to albumin and α-1 acid glycoprotein^46^. The epertinib concentration in brain metastases of the IVM with breast cancer is significantly higher than that of lapatnib. P-gp and BCRP, expressed at BTB, negatively impact the migration of TKIs to sites of brain metastasis. Epertinib has a high migration rate to brain metastases in the IVM, even though the free fraction in mouse plasma is low.

Co-localization of epertinib and heme B, suggests that tumor concentration of epertinib relates to blood volume around tumors and tumor HER2 expression levels. IMS enables us to analyze the tissue localization of drugs and endogenous molecules, and is useful in PK/PD and mechanistic efficacy and toxicity analyses of drugs^38,47^. Epertinib was reported to bind to an inactive conformation of enzyme and showed a slower off-rate of dissociation from EGFR and HER2 than lapatinib^13^. Comparison of the effects of EGFR and HER2 phosphorylation between 6 and 24 h after a single administration of epertinib revealed that the inhibitory activity of epertinib persisted at 24 h, while that of lapatinib had largely disappeared^13^. The sustained kinase inhibitory activity of epertinib may contribute to the superior antitumor activity observed *in vivo*.

Lapatinib has response rates in the brain range from 2.6 to 6% in heavily pre-treated patients^48,49^. However, when added to capecitabine, response rates increase to 20 to 33%^48,49^. Comparison of single agent lapatinib and epertinib concentrations in brain metastases of mice revealed that epertinib was detected at significantly higher concentrations than lapatinib. Additionally, epertinib, administered as a single agent showed antitumor activity against brain metastases in the presence of an intact BTB. Single agent epertinib showed sufficient antitumor efficacy against brain metastases, and it is assumed that combination with other chemotherapy, or HER2 targeting agents would increase potency. Recently, our colleague has reported a phase I/II combination study data for epertinib^18^. The objective response rate (ORR) when epertinib was administered in heavily pretreated HER2-positive metastatic breast cancer patients in combination with trastuzumab or in combination with trastuzumab and capecitabine was 67% and 56%, respectively. Compared to historical data for combination studies with trastzumab and lapatinib (ORR: 10.3%) or tucatinib (ONT-380) (ORR: 26%), epertinib showed better ORR^45,50^. Quantitative IMS showed that epertinib was transported into brain metastases in HER2-positive breast cancer. Epertinib would be the best TKI for use in patients with early stage HER2-positive breast cancer with brain metastasis. Furthermore, administration of epertinib with an antibody unable to penetrate into the central nervous system, increases the efficacy of epertinib in patients. It is hoped that, in future studies, epertinib efficacy will be demonstrated for many patients with HER2-positive breast cancer who are suffering with brain metastasis.

## Methods

### Chemicals and reagents

Epertinib hydrochloride, deuterated epertinib (epertinib-*d*_9_), and lapatinib ditosylate monohydrate were synthesized at Shionogi & Co., Ltd. (Osaka, Japan). Lapatinib**-** *d*_7_ dihydrochloride was purchased from Toronto Research Chemicals Inc. (Toronto, ON, Canada). High-performance LC-grade methanol, acetonitrile, and isopropanol were purchased from Kanto Chemicals Co., Inc. (Tokyo, Japan). 2,5-Dihydroxybenzoic acid (DHB) was obtained from Sigma-Aldrich (St. Louis, MO, USA). Trifluoroacetic acid (TFA) was obtained from Wako Pure Chemical Industries, Ltd. (Osaka, Japan). Ammonium hydrogen carbonate was purchased from Nacalai Tesque Inc. (Kyoto, Japan). Water was purified with a Milli-Q gradient-A10 purification system (Millipore, Bedford, MA, USA). Texas-Red^®^ dextran (molecular weight: 3 kDa) was purchased from Molecular Probes, Inc. (Eugene, OR, USA). Beetle luciferin, potassium salt was obtained from Promega Corporation (Madison, WI, USA). HercepTest^TM^ was purchased from Agilent Technologies (Santa Clara, CA, USA).

### Cells

The cell lines employed in this study were: NCI-H1975-luc (luciferase expressing lung cancer), MDA-MB-361 (breast cancer), MDA-MB-361-luc-BR2: BR2, and MDA-MB-361-luc-BR3: BR3 (luciferase expressing breast cancer with brain metastases). NCI-H1975 and MDA-MB-361 were purchased from American Type Culture Collection. Cell lines were propagated after resuscitation and cryopreserved within one month. NCI-H1975-luc, BR2, and BR3 were established by transfection of firefly luciferase expression vector into each cell type and subsequent cloning. For each study, cells were resuscitated and passaged for less than five months before use. The authenticity of luciferase expressing breast cancer cell lines was confirmed using short tandem repeat profiling (National Institute of Biomedical Innovation). NCI-H1975-luc was maintained in Dulbecco’s Modified Eagle’s Medium (DMEM) supplemented with 10% fetal bovine serum (FBS), 100 units/mL penicillin, 100 units/mL streptomycin, and 500 µg/mL G418. MDA-MB-361, BR2, and BR3 were maintained in DMEM with 20% FBS, 100 U/mL penicillin, 100 U/mL streptomycin, and 500 µg/mL G418.

### Animal experiments

All experimental protocols were approved by the Institutional Animal Care and Use Committee of Shionogi & Co., Ltd (Osaka, Japan) and performed in accordance with the Guidelines for Animal Experiments of Shionogi & Co., Ltd, which meet the ethical standards required by the law and the guidelines about the experimental animals in Japan. Nude mice (BALB/cAJcl-nu/nu, CLEA Japan) were used for *in vivo* studies. All mice were female and six to nine weeks old at the time of implantation. In pharmacokinetic studies, plasma and brain concentrations of epertinib or lapatinib were measured after a single oral administration of epertinib hydrochloride or lapatinib ditosylate monohydrate at a dose of 20 mg/kg (as epertinib or lapatinib) in nude mice implanted intracranially with MDA-MB-361 cells. In the MALDI-IMS study, epertinib hydrochloride (50 mg/kg or 100 mg/kg as epertinib) or lapatinib ditosylate monohydrate (50 mg/kg or 100 mg/kg as lapatinib) suspended in 0.5% methylcellulose was orally administrated to all mouse models. Mice were euthanized by isoflurane anesthesia and exsanguinated via the inferior vena cava 4 or 8 h after oral administration. Plasma was separated by centrifugation at 3,000 rpm for 15 min at 4 °C and stored at −80 °C until analysis. After blood sampling, brain tissues were immediately excised, frozen on dry ice, and stored at −80 °C prior to sectioning.

### Intraventricular and Intracranial implant mice models

In the experimental brain metastasis of breast cancer or lung cancer (IVM), a cell suspension containing 2.0  ×  10^6^ cells (BR2), 1.7  ×  10^6^ cells (BR3), or 4.0  ×  10^5^ cells (NCI-H1975-luc) was implanted into the left ventricle of anaesthetized mice (Supplementary Fig. S1). In the intracranial implantation mice model, the cell suspension containing 3.1  ×  10^5^ cells was implanted into the craniotomy region (2 mm to the left of the bregma and 1 mm anterior to the coronal suture). For measurement of bioluminescence, 0.2 mL of 10 mg/mL luciferin solution was injected intravenously into anesthetized mice via the tail vein and the photons emitted from tumor measured by IVIS Imaging System 200 (Caliper Life Sciences).

### Sample preparation for MALDI-IMS

Frozen brain tissues were cut into 10-µm sections using a cryostat (Leica CM3050 S; Leica Microsystems Inc., Wetzlar, Germany) at −20 °C. Sections were thaw-mounted onto glass microscope slides (Superfrost; Thermo Fisher Scientific Inc.) and stored at −80 °C until use. Epertinib hydrochloride, epertinib-*d*_9_ hydrochloride, and lapatinib ditosylate monohydrate were weighed and dissolved in methanol to prepare stock solutions (1 mmol/L). A stock solution of 0.1 mmol/L lapatinib**-** *d*_7_ dihydrochloride in methanol was prepared. For epertinib IMS analysis, a stock solution was diluted stepwise with 50% methanol to prepare standard solutions for calibration at concentrations from 15 to 15,000 nmol/L. A stock solution of lapatinib was serially diluted with supernatant in which blank rat plasma was mixed with an equal volume of methanol and centrifuged at 14,000 rpm for 3 min at 4 °C to prepare standard solutions at concentrations from 5 to 5,000 nmol/L. Sections were vacuum desiccated for 15 min at room temperature, and optical images were acquired by a scanner (Scanjet G4050; Hewlett-Packard Co., Palo Alto, CA, USA). Standard solutions were spotted onto the glass microscope slides at 1 µL. Epertinib-*d*_9_ (1 mmol/L) or lapatinib**-** *d*_7_ (0.1 mmol/L) were used as internal standards for epertinib or lapatinib, respectively. The matrix solution (30 mg/mL DHB dissolved in 1:1, v/v methanol-water containing 0.2% TFA) with the solution of epertinib-*d*_9_ (final concentration: 1 µmol/L) or lapatinib**-** *d*_7_ (final concentration: 0.1 µmol/L) was spray-coated all-over the glass slides using an ImagePrep^TM^ automated device with vibrational vaporization technology (Bruker Daltonics Inc., Billerica, MA, USA).

### MALDI-IMS analysis

Matrix-coated sections were analyzed using a linear ion trap mass spectrometer with a MALDI source (MALDI LTQ XL, Thermo Fisher Scientific Inc.) and a nitrogen laser (337 nm; 60 Hz). Laser energy and raster step size were set at 30 µJ and 100 µm, respectively. All compounds were detected in positive-ion mode using the product ion scans of their [M  +  H]^+^ ions. The collision energy was 60% and 70% of the maximum available energy required for the complete fragmentation of the precursor ion derived from Met-Arg-Phe-Ala peptide for epertinib/epertinib-*d*_9_ and lapatinib/lapatinib-*d*_7_, respectively^29^. The signal intensities of epertinib (*m/z* 560  →  461), epertinib-*d*_9_ (*m/z* 569  →  466), lapatinib (*m/z* 581  →  365), and lapatinib-*d*_7_ (*m/z* 588  →  365) within each region of interest were averaged. Heme B was detected by MS3 (*m/z* 616  →  557  →  498) as a blood biomarker^30^.

### LC/MS/MS analysis

To measure the concentration of epertinib or lapatinib in plasma samples, 500 µL of acetonitrile was added to 10 µL of the samples. The mixture was stirred for 10 min and centrifuged at 5,000 rpm for 5 min at 10 °C, and 1 µL of the supernatant was injected into the LC-MS/MS system. An ultra-HPLC system (Nexera LC-20A, Shimadzu Corp., Kyoto, Japan) with a triple quadrupole mass spectrometer (API 5000^TM^, AB Sciex, Forester City, CA, USA) was used for LC/MS/MS analysis. The LC-MS/MS system was controlled by Analyst 1.4.2 (AB Sciex) software. Chromatographic separation was performed using a Cadenza CD-C18 HT column (50  ×  2.0 mm i.d., 3 µm; Imtakt Corp., Kyoto, Japan). The mobile phase, consisting of (A) 10 mmol/L ammonium hydrogen carbonate for epertinib analysis or 10 mmol/L ammonium formate for lapatinib analysis and (B) acetonitrile, was pumped at a flow rate of 0.75 mL/min. Epertinib or lapatinib were eluted with a (B) 30-95% or 50-95% linear gradient, respectively. Selective reaction monitoring (SRM) transitions were *m/z* 561  →  338 at the collision energy of 40 eV and *m/z* 581  →  365 at the collision energy of 50 eV of epertinib and lapatinib, respectively. Using Analyst software, the concentration of epertinib or lapatinib was calculated from a calibration curve generated by analyzing a series of plasma samples from blank rats containing known quantities of epertinib or lapatinib.

### BTB permeability

For fluorescence analysis of BTB permeability in IVM, 0.2 mL of 10 mg/mL Texas- Red^®^ dextran solution was injected intravenously into each anesthetized mouse via the tail vein 5 min before euthanasia (Supplementary Fig. S1). Brain sections adjacent to those used for histological analyses were examined for fluorescence to capture images of Texas-Red^®^ fluorescence intensity (BZ-X700, Keyence). After image capture, using a fluorescence microscope, sections were fixed with 4% paraformaldehyde for 10 min and permeabilized with 0.3% Triton-X 100 for 10 min. To identify the position of the tumor in the brain section, sections were stained with 4′,6-diamidino-2-phenylindole, dihydrochloride (DAPI, ProLong^®^ Gold Antifade Mountant with DAPI, ThermoFisher Scientifc). Analysis of BTB permeability was performed by measuring Texas-Red^®^ fluorescence intensity within each region of brain metastases and for areas of normal brain and choroid plexus.

### Histological analysis

The sections adjacent to those used for MALDI-IMS studies were fixed in 10% neutral buffered formalin for 10 min and stained with hematoxylin and eosin (H&E) or giemsa. Stained sections were examined microscopically to identify the localization and distribution of tumors in the brain sections. HER2 immunohistochemistry (IHC) was performed as described in the HercepTest^TM^ instructions.

### Data analysis

Spectral data were acquired using Xcalibur 2.0.7 software (Thermo Fisher Scientific Inc.) and the localization and concentration of all compounds in the brain sections were analyzed using Quantinetix 1.7.10 software (ImaBiotech, Loos, France). The fluorescence intensity of Texas-Red^®^ dextran and immunostaining intensity of HER2 were analyzed using a BZ-X analyzer (Keyence). Statistical analyses were performed using SAS software (version 9.4).

## Electronic supplementary material

Supplementary information
