# Supplementary material for: Distribution analysis of epertinib in brain metastasis of HER2-positive breast cancer by imaging mass spectrometry and prospect for antitumor activity
Source: Sci Rep. 2018 Jan 10;8:343. doi: 10.1038/s41598-017-18702-2 (PMC5762859; doi:10.1038/s41598-017-18702-2)

Distribution analysis of epertinib in brain metastasis of HER2-positive breast cancer by imaging mass spectrometry and prospect for antitumor activity

Yukari Tanaka[1*], Michinari Hirata[2], Satomi Shinonome[2], Mikinori Torii[3], Ken-ichi Nezasa[1], and Hidekazu Tanaka[2]

[1]Drug Metabolism and Pharmacokinetics, Developmental Research Laboratories, Shionogi & Co., Ltd., Toyonaka, Osaka, Japan

[2]Oncology and Immunology, Drug Discovery and Disease Research Laboratories, Shionogi & Co., Ltd., Toyonaka, Osaka, Japan

[3]Drug Safety Evaluation, Developmental Research Laboratories, Shionogi & Co., Ltd., Toyonaka, Osaka, Japan

Supplementary information

Supplementary Table S1. Transcellular transport of [^{14}C]-epertinib, across Caco-2 cell monolayers at 37°C

Compound	Concentration (μmol/L)	P_{app} (×10^{-6} cm/sec)		P_{app} ratio
		Apical to basal	Basal to apical	
[^{14}C]-epertinib	1	1.17 ± 0.02	2.25 ± 0.14	1.9
	5	1.44 ± 0.05	1.83 ± 0.10	1.3
	20	1.69 ± 0.17	1.51 ± 0.08	0.9

Each value except the values of P_{app} ratio represents the mean ± SD of three samples.

Each value of P_{app} ratio was calculated by using the mean P_{app} value of three samples.

Supplementary Figure S1. Experimental brain metastasis of breast cancer (intraventricular mouse model): experimental method

Schematic showing the methodology of brain metastases development in mice, followed by drug and permeability tracer administration, and the equipment used to measure lesion permeability and distribution.

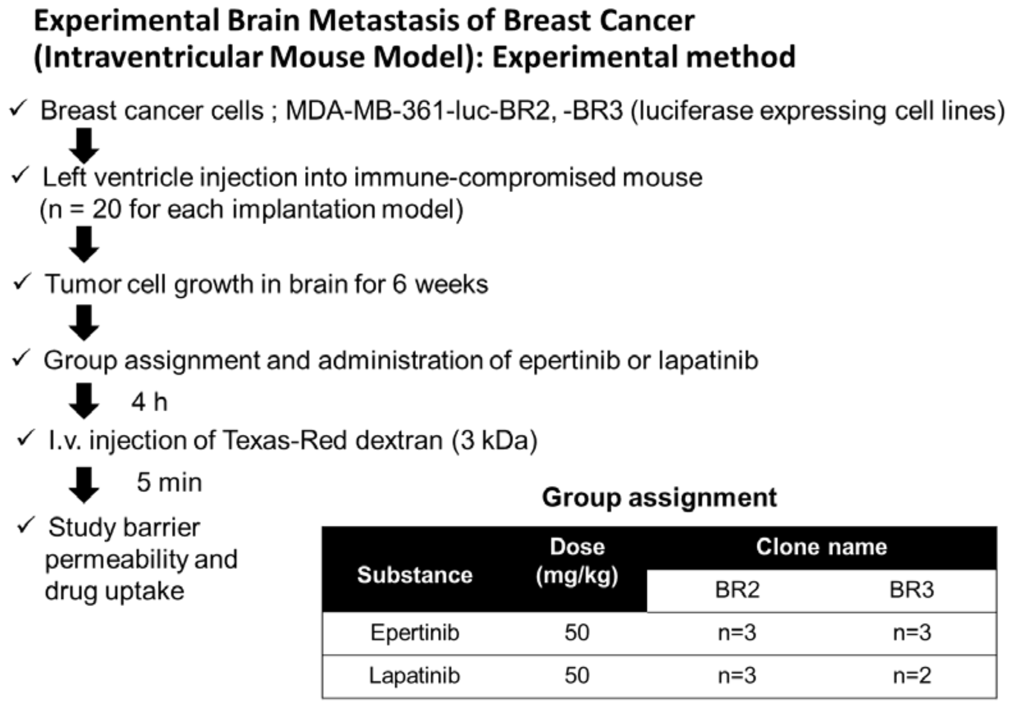

Substance	Dose (mg/kg)	Clone name	
		BR2	BR3
Epertinib	50	n=3	n=3
Lapatinib	50	n=3	n=2

Supplementary Figure S2. Distribution of epertinib and lapatinib in brain metastases of the breast cancer IVM

Representative images of mouse brain sections showing BR2 IVM with breast cancer with at 4 h after oral administration of epertinib hydrochloride or lapatinib ditosylate monohydrate at a dose of 50 mg/kg (as epertinib (**A**) or lapatinib (**B**)). The magnified views are shown in upper row: H&E images, middle row: ion images of epertinib or lapatinib, and lower row: ion images of epertinib or lapatinib co-registered with H&E. Typical images of mouse brain sections showing diamidino phenylindole fluorescence (**C**), Texas-Red® dextran (3 kDa) fluorescence (**D**), and HER2 IHC staining (**E**) from an animal with BR2 brain metastases. Arrows indicate the position of tumors and the position of choroid plexus. Dashed lines of black and yellow: the region of tumors (**A and B**), white: the region of brain parenchyma (**D**).

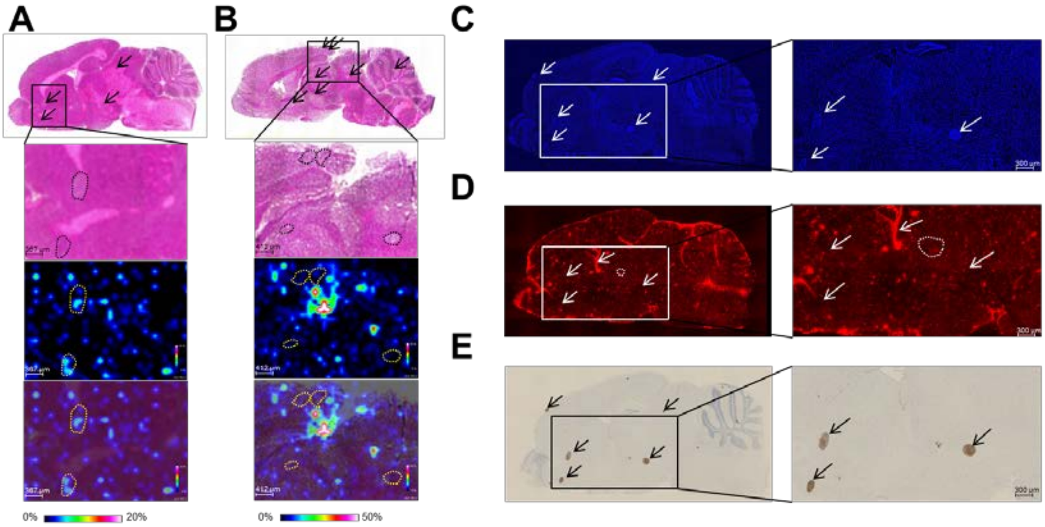

Supplementary Figure S3. Concentration of radioactivity and epertinib in plasma after single oral administration of [^{14}C]-epertinib hydrochloride to non-fasting male rats (dose: 5 mg as epertinib/kg)

Data are expressed as the mean values + SD of four animals.

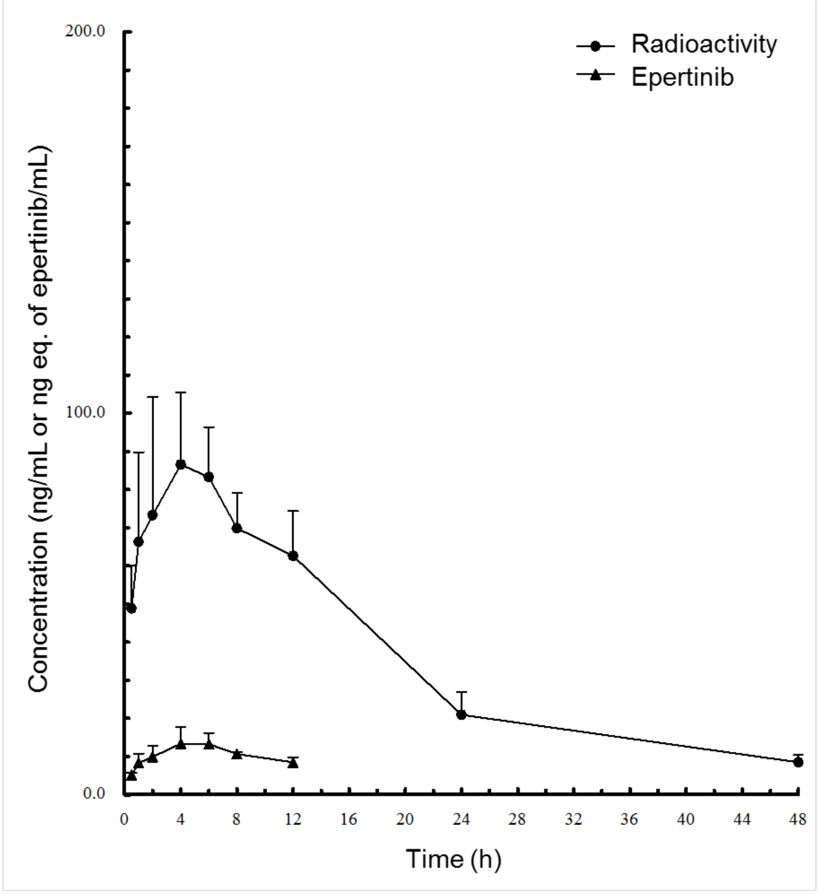

Supplementary Figure S4. Representavive calibration curves of epertinib and lapatinib measured by IMS

Representative data for calibration curves of epertinib (**A**) and lapaitnib (**B**)

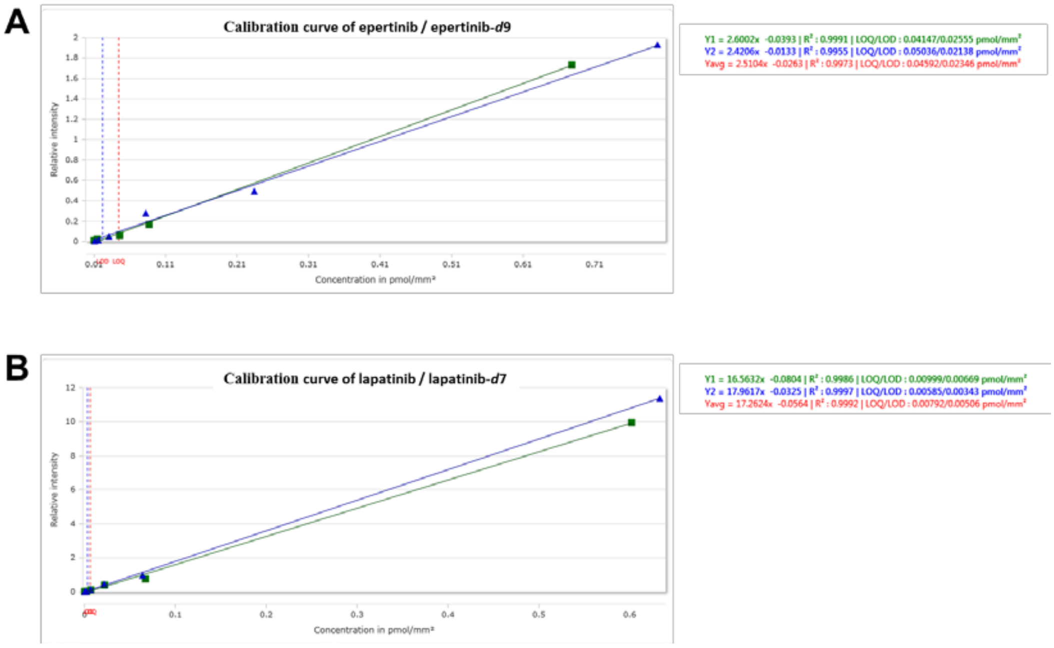

Supplement: Supplementary file 1 — Supplementary information [file 41598_2017_18702_MOESM1_ESM.pdf]
